# The Effect of Small Doses of Fructose and Its Epimers on Glycemic Control: A Systematic Review and Meta-Analysis of Controlled Feeding Trials

**DOI:** 10.3390/nu10111805

**Published:** 2018-11-20

**Authors:** Jarvis C. Noronha, Catherine R. Braunstein, Sonia Blanco Mejia, Tauseef A. Khan, Cyril W. C. Kendall, Thomas M. S. Wolever, Lawrence A. Leiter, John L. Sievenpiper

**Affiliations:** 1 Toronto 3D (Diet, Digestive Tract, and Disease) Knowledge Synthesis and Clinical Trials Unit, Clinical Nutrition and Risk Factor Modification Centre, St. Michael’s Hospital, Toronto, ON M5C 2T2, Canada; jarvis.noronha@mail.utoronto.ca (J.C.N.); catherine.braunstein@mail.utoronto.ca (C.R.B.); sonia.blancomejia@mail.utoronto.ca (S.B.M.); tauseef.khan@utoronto.ca (T.A.K.); cyril.kendall@utoronto.ca (C.W.C.K.); thomas.wolever@utoronto.ca (T.M.S.W.); leiterl@smh.ca (L.A.L.); 2 Department of Nutritional Sciences, Faculty of Medicine, University of Toronto, Toronto, ON M5S 1A8, Canada; 3 College of Pharmacy and Nutrition, University of Saskatchewan, Saskatchewan, SK S7N 2Z4, Canada; 4 Li Ka Shing Knowledge Institute, St. Michael’s Hospital, Toronto, ON M5B 1T8, Canada; 5 Division of Endocrinology and Metabolism, St. Michael’s Hospital, Toronto, ON M5C 2T2, Canada; 6 Department of Medicine, University of Toronto, ON M5G 2C4, Canada

**Keywords:** fructose, allulose, tagatose, sorbose, catalytic dose, glycemia, HbA_1c_, glucose, insulin, meta-analysis

## Abstract

**Objective:** Contrary to the concerns that fructose may have adverse metabolic effects, an emerging literature has shown that small doses (≤10 g/meal) of fructose and its low-caloric epimers (allulose, tagatose, and sorbose) decrease the glycemic response to high glycemic index meals. Whether these acute reductions manifest as sustainable improvements in glycemic control is unclear. Our objective was to synthesize the evidence from controlled feeding trials that assessed the effect of small doses of fructose and its low-caloric epimers on glycemic control. **Methods:** We searched MEDLINE, EMBASE, and the Cochrane Library through April 18, 2018. We included controlled feeding trials of ≥1 week that investigated the effect of small doses (≤50 g/day or ≤10% of total energy intake/day) of fructose and its low-caloric epimers on HbA_1c_, fasting glucose, and fasting insulin. Two independent reviewers extracted data and assessed risk of bias. Data were pooled using the generic inverse variance method and expressed as mean differences (MDs) with 95% confidence intervals (CIs). Heterogeneity was assessed using the Cochran Q statistic and quantified using the *I*^2^ statistic. Grading of Recommendations Assessment, Development and Evaluation (GRADE) assessed the certainty of the evidence. **Results:** We identified 14 trial comparisons (*N* = 337) of the effect of fructose in individuals with and without diabetes, 3 trial comparisons (*N* = 138) of the effect of allulose in individuals without diabetes, 3 trial comparisons (*N* = 376) of the effect of tagatose mainly in individuals with type 2 diabetes, and 0 trial comparisons of the effect of sorbose. Small doses of fructose and tagatose significantly reduced HbA_1c_ (MD = −0.38% (95% CI: −0.64%, −0.13%); MD = −0.20% (95% CI: −0.34%, −0.06%)) and fasting glucose (MD = −0.13 mmol/L (95% CI: −0.24 mmol/L, −0.03 mmol/L)); MD = −0.30 mmol/L (95% CI: −0.57 mmol/L, −0.04 mmol/L)) without affecting fasting insulin (*p* > 0.05). Small doses of allulose did not have a significant effect on HbA_1c_ and fasting insulin (*p* > 0.05), while the reduction in fasting glucose was of borderline significance (*p* = 0.05). The certainty of the evidence of the effect of small doses of fructose and allulose on HbA_1c_, fasting glucose, and fasting insulin was graded as low. The certainty of the evidence of the effect of tagatose on HbA_1c_, fasting glucose, and fasting insulin was graded as moderate. **Conclusions:** Our results indicate that small doses of fructose and tagatose may improve glycemic control over the long term. There is a need for long-term randomized controlled trials for all four sugars to improve our certainty in the estimates.

## 1. Introduction

Sugar has emerged as a major nutrient of concern in diabetes, creating a need for caloric and low-caloric sugar alternatives. The fructose moiety of sugar has been implicated as a potent driver of type 2 diabetes due to its unique set of biochemical, metabolic, and endocrine responses [1,2,3]. This is largely based on the results of ecological observations, animal models of fructose overfeeding, and select human studies assessed in isolation. The important biological mechanisms by which small doses of fructose may assist in the hepatic handling of glucose have largely been ignored. Contrary to the concerns that fructose may have adverse metabolic effects, an emerging literature suggests that small doses (≤10 g/meal) of fructose and its low-caloric epimers (allulose, tagatose, and sorbose) may, in fact, improve the metabolic handling of glucose. 

Fructose exists in foods either as free fructose (e.g., fruits, honey, or high-fructose corn syrup) or fructose bound to glucose (sucrose) [4]. Allulose, the c-3 epimer of fructose, is a low-calorie sugar (~0.4 kcal/g) found naturally in small amounts in dried fruits, brown sugar, and maple syrup [5]. Tagatose, the c-4 epimer of fructose, is also a low-calorie sugar (~1.5 kcal/g) found naturally in small amounts mainly in dairy products (e.g., milk, cheese, and yogurt) [6,7]. Sorbose, the c-5 epimer of fructose, is the least studied of the three epimers and its presence in foods is currently unknown. 

Acute clinical evidence has demonstrated that small doses (≤10 g/meal) of fructose, allulose, and tagatose decrease the postprandial glycemic response to high glycemic index meals (oral glucose, maltodextrins, mashed potatoes, and sandwiches) by 4–30% in healthy individuals and those with prediabetes or type 2 diabetes [8,9,10,11,12,13,14,15]. These acute effects have been shown to be sustainable over the long term in the case of fructose. A systematic review and meta-analysis of controlled feeding trials showed that small doses (defined as ≤36 g/day based on three meals at ≤ 10g/meal and two snacks at ≤3 g/snack) of fructose in exchange for other carbohydrates (mainly starch) decreased HbA_1c_ by 0.4% [16]. Whether the acute benefits of allulose and tagatose translate into meaningful reductions in long-term glycemic control remains unclear.

Our objective was to update and expand the previous systematic review and meta-analysis by synthesizing the current available evidence of the effect of small doses of fructose and its low-caloric epimers (allulose, tagatose, and sorbose) on glycemic control. 

## 2. Materials and Methods

This systematic review and meta-analysis was conducted in accordance with the Cochrane Handbook for Systematic Reviews and Interventions [17] and reported in accordance with the Preferred Reporting Items for Systematic Reviews and Meta-analyses (PRISMA) guidelines [18]. The study protocol is registered at ClinicalTrials.gov (identifier: NCT02776722). 

### 2.1. Data Sources and Searches 

We searched MEDLINE, EMBASE, and Cochrane Central Register of Controlled Trials through April 18, 2018 for relevant articles. The full search terms used in this study are presented in Table S1. To limit the database searches to controlled trials, validated filters from McMaster University Health Information Research unit were applied [19]. Manual searches supplemented the electronic search strategy. Reference lists of selected studies and reviews were also searched to identify additional articles. 

### 2.2. Study Selection 

We included controlled feeding trials (randomized and nonrandomized) conducted in humans lasting ≥1 week that investigated the effect of small doses (≤50 g/day or ≤10% of total energy intake/day) of fructose, allulose, tagatose, and sorbose on HbA_1c_, fasting glucose, and fasting insulin. The ≤50 g/day or ≤10% of total energy intake/day dose threshold for chronic feeding allowed for the intake of fructose and its low-caloric epimers as part of three main meals (≤10 g/meal) and three snacks (≤5 g/meal) per day and aligned with current guidelines not to exceed 10% energy from free or added sugars [20,21]. Trials that lasted <1 week, administered fructose and its epimers intravenously, lacked an adequate comparator, or did not provide suitable endpoint data were excluded. 

### 2.3. Data Extraction 

Two reviewers (J.C.N and C.R.B) independently reviewed and extracted relevant data on trial characteristics and outcomes from each report. Any discrepancies were reconciled by consensus. Study authors were contacted for missing outcome data when it was indicated that an outcome was measured but not reported. Mean differences (MDs) and standard errors (SEs) of the MDs between the treatment arm and comparator arm were extracted as the main endpoints for each outcome. Between treatment change-from-baseline differences (MDs and SEs) were preferred over end differences (MDs and SEs) as the primary endpoint. If trials did not report these values, we calculated them from available data using statistics or imputed them using standard formulas [17]. In the absence of numerical values for outcome measurements or inability to contact study authors, values were extracted from Plot Digitizer where available [22]. When multiple comparators were present, starch or glucose were preferred to minimize the influence of heterogeneity which would be present when combining arms. Risk of bias of the included studies was assessed using the Cochrane Collaboration Risk of Bias Tool [23]. 

### 2.4. Data Synthesis and Analysis 

Data analyses were conducted using Review Manager (RevMan) version 5.3 (The Nordic Cochrane Centre, The Cochrane Collaboration, 2014, Copenhagen, Denmark) for primary analyses and Stata version 13 (Stata Corp LP, College Station, TX, USA) for subgroup and publication bias analyses. A generic inverse-variance method with random-effects models was used to calculate the pooled mean differences and 95% confidence intervals (CIs). Random-effects models were used even in the absence of statistically significant interstudy heterogeneity, as they typically yield more conservative estimates. Fixed-effect models were used when fewer than five trials were available for an outcome. For each outcome, data were expressed as MD with 95% CIs and standardized mean difference (SMD) with 95% CIs. 

Interstudy heterogeneity was tested using the Cochran Q statistic (significance at *P_Q_* < 0.10) and quantified with the *I*^2^ statistic, where *I*^2^ > 50% and *P_Q_* < 0.10 was evidence of substantial heterogeneity. As a sensitivity analysis, we removed each study within a given outcome and recalculated the summary effect using the “leave one out” approach [24]. Sensitivity analyses were also conducted using different correlation coefficient values for crossover trials (0.25 and 0.75) to test for robustness of the effect size. If more than 10 trials were available for a given outcome, sources of heterogeneity were explored using *a priori* subgroup analyses by age, comparator, dose, sugar form, study design, duration, energy balance, and risk of bias [25,26,27]. If more than 10 trials were available for a given outcome, the possibility of publication bias was explored by inspecting funnel plots and conducting Egger’s and Begg’s tests (each significant at *p* < 0.10). If publication bias was suspected, results were shown without imputation and with “missing” studies imputed with Duval and Tweedie’s trim and fill method [28]. 

### 2.5. Grading of Recommendations, Assessment, Development, and Evaluation (GRADE) Assessment 

The certainty of the evidence was assessed using the GRADE approach [29,30,31,32,33,34,35,36,37,38,39,40,41]. Evidence was graded as high, moderate, low, or very low quality. Controlled feeding trials were graded as high-quality evidence by default and downgraded based on five prespecified criteria: (1) risk of bias (assessed through the Cochrane Risk of Bias tool); (2) inconsistency (substantial unexplained interstudy heterogeneity, *I*^2^ > 50%, and *P_Q_* < 0.10); (3) indirectness (presence of factors that limited the generalizability of the results); (4) imprecision (95% CIs for the effect estimates were wide or crossed the minimally important difference for benefit); and (5) publication bias (significant evidence of small-study effects). 

## 3. Results

### 3.1. Search Results

**Figure 1:**
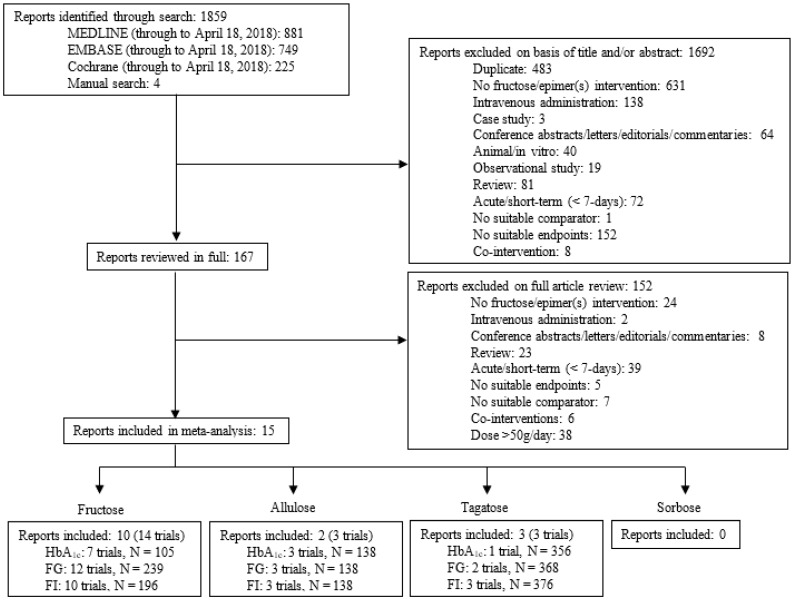
Literature search.

Figure 1 illustrates the systematic search and selection of literature. We identified 1859 reports of which 15 met the eligibility criteria [11,42,43,44,45,46,47,48,49,50,51,52,53,54,55,56,57]. Of the 15 eligible reports, 10 reports (14 trial comparisons, *N* = 337) assessed the effect of small doses of fructose [42,43,44,45,46,47,48,49,50,51,52,53], 2 reports (3 trial comparisons, *N* = 138) assessed the effect of small doses of allulose [11,54], and 3 reports (3 trial comparisons, *N* = 376) assessed the effect of small doses of tagatose [55,56,57] on glycemic control. No reports were identified that assessed the effect of small doses of sorbose on glycemic control.

### 3.2. Trial Characteristics

Table 1 shows the characteristics of the included trials.

#### 3.2.1. Characteristics of the Fructose Trials 

All of the fructose trials were randomized. Of the 14 trials, 7 trials were conducted in Europe, 6 trials were conducted in North America, and 1 trial was conducted in Israel. Trials were conducted in individuals with hypertriglyceridemia (1/14), type 1 diabetes (2/14), type 2 diabetes (2/14), those who were healthy (2/14), overweight/obese (4/14), mixed populations of lean and overweight/obese individuals (2/14), and individuals with type 1 and type 2 diabetes (1/14). Trial sizes tended to be small (median *N* = 21 participants) and short (median follow-up duration = 2.5 weeks; range 1–52 weeks). Fructose was administered in liquid or mixed forms at a median dose of 36 g/d (7.2% daily energy intake). Starch (6/14), glucose (6/14), and dextromaltose (2/14) were the comparators. 

#### 3.2.2. Characteristics of the Allulose Trials 

All of the allulose trials were randomized and were conducted in Asia (two trials in Korea and one trial in Japan). Trials were conducted in healthy individuals (1/3) and those who were overweight/obese (2/3). Allulose was administered in liquid form at a median dose of 14 g/d and the follow-up duration across all trials was 12 weeks. Sucralose (2/3 trials) and glucose (1/3 trials) were the comparators.

#### 3.2.3. Characteristics of the Tagatose Trials

Two of the three tagatose trials were randomized, and one was nonrandomized. Of the three trials, two trials were conducted in Europe (Switzerland and Denmark), and the one multicenter trial was conducted in the United States and India. Trials were conducted in healthy participants (2/3) and those with type 2 diabetes (1/3). Across all three trials, tagatose was provided in either solid, liquid, or mixed form at a median dose of 45 g/day with a follow-up duration of 2–40 weeks. Sucrose (2/3) and Splenda® (1/3) were the comparators.

### 3.3. Risk of Bias 

Figure S1 shows the summary Cochrane Risk of Bias tool assessments of the effect of fructose, allulose, and tagatose on glycemic control. No serious risk of bias was detected.

### 3.4. The Effect of Small Doses of Fructose on Glycemic Control 

#### 3.4.1. HbA_1c_

**Figure 2:**
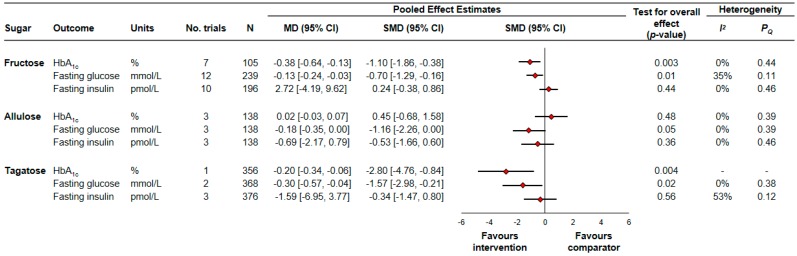
Summary of the pooled effect estimates from controlled feeding trials assessing the effect of small doses of fructose, allulose, and tagatose on glycemic control. To allow the summary estimates for each endpoint to be displayed on the same axis, mean differences (MDs) were transformed to standardized mean differences (SMDs) and pseudo-95% confidence intervals (CIs), which were derived directly from the original mean difference and 95% CI. N, number of participants.

Figure 2 and Figure S2 show the effect of small doses of fructose on HbA_1c_. Small doses of fructose significantly reduced HbA_1c_ (7 trial comparisons, *N* = 105, MD = −0.38% (95% CI −0.64% to −0.13%), *p* = 0.003) with no evidence of interstudy heterogeneity (*I*^2^ = 0%, *P_Q_* = 0.44).

#### 3.4.2. Fasting Glucose

Figure 2 and Figure S3 show the effect of small doses of fructose on fasting glucose. Small doses of fructose significantly reduced fasting glucose (12 trial comparisons, *N* = 239, MD = −0.13 mmol/L (95% CI −0.24 mmol/L to −0.03 mmol/L), *p* = 0.01) with no evidence of interstudy heterogeneity (*I*^2^ = 35%, *P_Q_* = 0.11). 

#### 3.4.3. Fasting Insulin

Figure 2 and Figure S4 show the effect of small doses of fructose on fasting insulin. Small doses of fructose did not have a significant effect on fasting insulin (10 trial comparisons, *N* = 196, MD = 2.72 pmol/L (95% CI −4.19 pmol/L to 9.62 pmol/L), *p* = 0.44). There was no evidence of interstudy heterogeneity (*I*^2^ = 0%, *P_Q_* = 0.46).

### 3.5. The Effect of Small Doses of Allulose on Glycemic Control 

#### 3.5.1. HbA_1c_

Figure 2 and Figure S5 show the effect of small doses of allulose on HbA_1c_. Small doses of allulose did not have a significant effect on HbA_1c_ (3 trial comparisons, *N* = 138, MD = 0.02% (95% CI −0.03% to 0.07%), *p* = 0.48). There was no evidence of interstudy heterogeneity (*I*^2^ = 0%, P_Q_ = 0.39). 

#### 3.5.2. Fasting Glucose

Figure 2 and Figure S6 show the effect of small doses of allulose on fasting glucose. Small doses of allulose reduced fasting glucose, though the effect was of borderline significance (3 trial comparisons, *N* = 138, MD = −0.18 mmol/L (95% CI −0.35 mmol/L to 0.00 mmol/L), *p* = 0.05). There was no evidence of interstudy heterogeneity (*I*^2^ = 0%, *P_Q_* = 0.39). 

#### 3.5.3. Fasting Insulin

Figure 2 and Figure S7 show the effect of small doses of allulose on fasting insulin. Small doses of allulose did not have a significant effect on fasting insulin (3 trial comparisons, *N* = 138, MD = −0.69 pmol/L (95% CI −2.17 pmol/L to 0.79 pmol/L), *p* = 0.36). There was no evidence of interstudy heterogeneity (*I*^2^ = 0%, *P_Q_* = 0.46). 

### 3.6. The Effect of Small Doses of Tagatose on Glycemic Control 

#### 3.6.1. HbA_1c_

Figure 2 and Figure S8 show the effect of small doses of tagatose on HbA_1c_. Small doses of tagatose significantly reduced HbA_1c_ (1 trial comparison, *N* = 356, MD = −0.20% (95% CI −0.34% to −0.06%), *p* = 0.004). Interstudy heterogeneity was not assessed due to the availability of only one trial. 

#### 3.6.2. Fasting Glucose

Figure 2 and Figure S9 show the effect of small doses of tagatose on fasting glucose. Small doses of tagatose significantly reduced fasting glucose (2 trial comparisons, *N* = 368, MD = −0.30 mmol/L (95% CI −0.57 mmol/L to −0.04 mmol/L), *p* = 0.02) with no evidence of interstudy heterogeneity (*I*^2^ = 0%, *P_Q_* = 0.38). 

#### 3.6.3. Fasting Insulin

Figure 2 and Figure S10 show the effect of small doses of tagatose on fasting insulin. Small doses of tagatose did not have a significant effect on fasting insulin (3 trial comparisons, *N* = 376, MD = −1.59 pmol/L (95% CI −6.95 pmol/L to 3.77 pmol/L), *p* = 0.56). There was no evidence of interstudy heterogeneity (*I*^2^ = 53%, *P_Q_* = 0.12). 

### 3.7. Sensitivity Analyses 

Table S2 shows select sensitivity analyses in which the systematic removal of individual trials altered results. No one trial modified the significance, direction, or magnitude of the pooled estimates or changed the significance for heterogeneity for HbA_1c_, fasting glucose, and fasting insulin in the fructose analysis. In the pooled analysis of the effect of allulose on fasting glucose, the removal of Hayashi et al. [11] altered the results from borderline significant (*p* = 0.05) to statistically significant (*p* < 0.05). In the pooled analysis of the effect of tagatose on fasting glucose, the removal of Ensor et al. [58] altered the results from statistically significant to nonsignificant. 

Table S3 shows sensitivity analyses in which we used different correlation coefficients (0.25 and 0.75) for paired analyses of crossover trials. In the pooled analysis of the effect of fructose on fasting glucose, the use of 0.75 altered the results from significant to nonsignificant. In the pooled analysis of the effect of tagatose on fasting insulin, the use of 0.75 altered the heterogeneity from nonsignificant to significant. 

### 3.8. Subgroup Analyses 

We were only able to conduct *a priori* subgroup analyses of the effect of small doses of fructose on fasting glucose (Figures S11 and S12) and fasting insulin (Figures S13 and S14). Subgroup analyses of the effect of small doses of fructose on HbA_1c_ and of the effect of small doses of allulose and tagatose on HbA_1c_, fasting glucose, and fasting insulin could not be assessed, owing to <10 trial comparisons. 

There was evidence of significant effect modification by health status (*p* = 0.03) and fructose form (*p* = 0.02) of the effect of small doses of fructose on fasting glucose (Figures S3 and S11). There was no evidence of significant effect modification in any of the other subgroups. 

### 3.9. Publication Bias Analyses 

Figure S15 shows the funnel plots of the effect of small doses of fructose on fasting glucose and fasting insulin where ≥10 trials were available. There was no evidence of funnel-plot asymmetry. Formal testing with the Egger’s and Begg’s tests did not show evidence of small-study effects (*p* > 0.05 for both). 

### 3.10. GRADE Assessment 

Table S4 shows the GRADE assessment summary of the effect of small doses of fructose, allulose, and tagatose on glycemic control. The evidence of the effect of small doses of fructose and allulose on HbA_1c_, fasting glucose, and fasting insulin was graded as low quality owing to downgrades for serious imprecision and serious indirectness. The evidence of the effect of tagatose on HbA_1c_, fasting glucose, and fasting insulin was graded as moderate quality owing to downgrades for serious imprecision. 

## 4. Discussion

### 4.1. Summary of Findings 

The present systematic review and meta-analysis was conducted to assess the effect of small doses of fructose and its low-caloric epimers (allulose, tagatose, and sorbose) on glycemic control. We identified 14 trial comparisons (*N* = 337) of the effect of fructose in individuals with and without diabetes, 3 trial comparisons (*N* = 138) of the effect of allulose in individuals without diabetes, 3 trial comparisons (*N* = 376) of the effect of tagatose mainly in individuals with type 2 diabetes, and 0 trial comparisons of the effect of sorbose. Small doses of fructose and tagatose significantly reduced HbA_1c_ and fasting glucose, without affecting fasting insulin. Small doses of allulose did not have a significant effect on HbA_1c_ and fasting insulin, while the reduction in fasting glucose was of borderline significance. 

### 4.2. Findings in the Context of Previous Research

The results from our fructose analysis are consistent with a previous meta-analysis that showed a beneficial effect of small doses of fructose (22.5–36 g/day) in isocaloric exchange for other carbohydrates (mainly starch) on markers of glycemic control [16]. Two previously conducted meta-analyses have also reported a beneficial effect of fructose in isocaloric exchange for other carbohydrates on glycated blood proteins in people with diabetes and without diabetes [58,59]. These findings are also consistent with a recent meta-analysis that demonstrated a significant reduction in HbA_1c_ and fasting glucose when fructose (mean dose, 68 g/day; range: 40–150 g/day) was substituted with glucose or sucrose-sweetened foods [60]. 

The results from our tagatose analysis are partly in alignment with previous uncontrolled clinical trials. When eight individuals with type 2 diabetes consumed 15 g of tagatose three times daily with food for one year, their HbA_1c_ concentrations fell from 10.6% to 9.6%, though the results were nonsignificant [61]. In another study, 5 g and 7.5 g of tagatose consumption three times daily with food for six months reduced HbA_1c_ by 0.1% and 0.2%, respectively, in 145 individuals with type 2 diabetes, though the results were nonsignificant [62]. 

### 4.3. Potential Mechanism(s) of Action 

One potential mechanism to explain the observed improvements in glycemic control is enhancement of hepatic glucokinase activity when small doses of fructose and tagatose are consumed with carbohydrates. Hepatic glucokinase is inhibited by glucokinase regulatory protein (GKRP) and this action is enhanced in the presence of fructose-6-phosphate [63,64,65,66,67,68]. Under fasting conditions, hepatic glucokinase is localized primarily in the nucleus, where it is bound to GKRP and fructose-6-phosphate [63,69,70]. In the postprandial state (presence of fructose or tagatose with glucose), fructose and tagatose are phosphorylated by ketohexokinase to fructose-1-phsophate and tagatose-1-phosphate, respectively [71,72]. These metabolites compete with fructose-6-phosphate binding to GKRP, and in doing so, release glucokinase from GKRP [73,74,75,76]. This enables the liberated and activated glucokinase to translocate from the nucleus to the cytosol, where it can drive hepatic glucose uptake, promote glycogen synthesis, suppress hepatic glucose output, and reduce plasma glucose concentrations. In the case of fructose, this mechanism has shown to relate to an ~30% reduction in hepatic glucose output under hyperglycemic conditions in people with type 2 diabetes [77] and ~3-fold increase in glycogen synthesis by ^13^C NMR spectroscopy under euglycemic conditions in people without diabetes [78]. It has also been shown that small doses of fructose reverse hepatic glucose sensing impairment in individuals with impaired fasting glucose [79]. In the case of tagatose, experiments on isolated rat hepatocytes have demonstrated an increased activity of glycogen synthase when tagatose was added to the medium [80]. Animal studies have also demonstrated an increase in hepatic glycogen content under chronic tagatose feeding conditions [81,82]. 

### 4.4. Implications

The present systematic review and meta-analysis demonstrated a 0.38% reduction in HbA_1c_ when small doses of fructose were consumed in isocaloric comparison with other carbohydrates. This reduction is clinically meaningful as it exceeds the threshold of ≥0.3% proposed by the U.S. Food and Drug Administration for the development of new drugs for diabetes and lies at the lower limit of efficacy expected for oral antihyperglycemic agents [83,84]. 

A benefit from small doses of fructose may be achieved through the consumption of low-glycemic index fruit (e.g., an apple has ~9–10 g of fructose) [85]. A secondary analysis of a randomized controlled trial in 152 participants with type 2 diabetes found that when comparing the highest with the lowest quartile of low-glycemic index fruit intake, the % change in HbA1c was reduced by ~0.5% [86]. This reduction is similar to what we found in our analysis, even though fructose was mainly consumed as crystalline fructose rather than in the form of low-glycemic index fruit. 

Although many of the studies in this meta-analysis assessed the effect of exchanging one sweetener for another, the use of fructose and tagatose as alternative sweeteners could be part of a broader strategy to decrease the intake of excess calories from all sugars and refined starches while promoting the intake of more nutrient-dense foods that are high in whole grains, viscous fibers, fruit and vegetables, pulses, nuts, and dairy or nondairy products. 

### 4.5. Consideration of a Dose Threshold for Harm 

Although it appears that fructose in isocaloric exchange for other carbohydrates may benefit glycemia, a dose threshold for harm must also be considered. A meta-analysis previously showed that fructose at doses >60 g/day (in excess of Diabetes Canada recommendations) or >10% energy in isocaloric exchange for carbohydrate increased serum triglyceride concentrations in individuals with type 2 diabetes [87]. Despite showing a tendency for improvement in HbA_1c_, another meta-analysis showed a consistent triglyceride-raising effect of fructose at doses >100 g/day (>95th percentile total US fructose intake) across different subjects [61]. This suggests that fructose may lead to unintended harm insofar as the excess calories it provides, and rather than focusing solely on fructose, it should be considered in the context of the whole diet. 

A dose threshold for harm of tagatose must also be considered. The presence of chronically elevated plasma uric acid concentrations (i.e., hyperuricemia) is a known risk factor for the development of gout. Ingestion of single high bolus doses (≥30 g) of tagatose has been associated with a mild, transient increase in plasma uric acid concentration in both healthy individuals with those with type 2 diabetes [72,88,89]. 

Gastrointestinal disturbances have also been reported with large single doses (>25 g) of fructose and tagatose intake in some individuals due to malabsorption of the sugars. In three separate studies, when the fructose dose was increased from 25 to 50 g in 10% solution, the prevalence of malabsorption increased from 0% to 37.5%, 11% to 59%, and 50% to 80%, respectively [90,91,92]. This malabsorption of fructose can be largely overcome by co-ingestion with glucose in the form of sucrose or high-fructose corn syrup [90,91,92,93]. A single-dose tolerance study in 73 young healthy male participants reported that consumption of 30 g tagatose led to nausea and diarrhea in 15.1% and 31.5% of participants, respectively [94]. Another tolerance study in eight healthy subjects and eight individuals with type 2 diabetes reported diarrhea, nausea, and/or flatulence in 100% of participants after consuming a single 75-g dose of tagatose [12]. Unlike fructose, it is unclear whether malabsorption of tagatose can be overcome by co-ingestion with glucose. 

### 4.6. Strengths and Limitations 

The present systematic review and meta-analysis has several strengths, including: (1) a rigorous search and selection process of available literature examining the effect of small doses of fructose and its low-caloric epimers (allulose, tagatose, and sorbose) on glycemic control; (2) inclusion of controlled feeding trials which gave us greater protection against bias; (3) the pooled synthesis of data from 20 trials involving 851 participants; and (4) an assessment of the overall certainty of the evidence using the GRADE assessment tool. However, some limitations also complicate the interpretation of these pooled analyses, including: (1) serious indirectness of the effect of small doses of fructose on HbA_1c_, fasting glucose, and fasting insulin, as most of the trials were of relatively short duration (median follow-up duration = 2 weeks). It is possible that the shorter trials may have underestimated the HbA_1c_ reduction given the evidence that HbA_1c_ reduces at ~0.1% per day at a steady state with a half-life of 5 weeks [95]; (2) serious indirectness of the effect of small doses of allulose on HbA_1c_, fasting glucose, and fasting insulin, as all of the available trials were conducted in Asia (Japan and Korea), which may affect the generalizability of the results; (3) serious imprecision of the effect of small doses of fructose, allulose, and tagatose on HbA_1c_, fasting glucose, and fasting insulin, as the 95% CI of the pooled effect estimates crossed the clinically meaningful threshold for benefit; and (4) the small number of available trials of the effect of allulose and tagatose meant that we were unable to conduct subgroup and publication bias analyses for any outcome related to these sugars. 

Balancing these strengths and limitations, the GRADE approach assessed the overall certainty of the available evidence as moderate for the effect of tagatose on HbA_1c_, fasting glucose, and fasting insulin and as low for the effect of fructose and allulose on HbA_1c_, fasting glucose, and fasting insulin. 

## 5. Conclusions

Our synthesis of the available evidence from controlled feeding trials demonstrates that small doses of fructose and tagatose modestly improve glycemic control in individuals with and without diabetes. Allulose failed to demonstrate such improvements, although data were limited to individuals without diabetes. Our confidence in the pooled estimates for these outcomes is moderate to low. Sources of uncertainty include imprecision of the effect of fructose, allulose, and tagatose and indirectness of the effect of fructose and allulose. More research is likely to have an important influence on our confidence in the pooled estimates. Further large, high-quality, randomized controlled trials of >6 months in various settings are needed to better understand the role of fructose and its low-caloric epimers in glycemic control. 

## Figures and Tables

**Table 1:** Characteristics of included trials.

Study	Subjects	Mean Age (SD or Range)	Setting	Design	Feeding Control *	Randomized	Dose (g/d) ^†^	Form ^‡^	Comparator	Background Diet **	Energy Balance	Follow-up Duration	Funding ***
**FRUCTOSE**													
Turner et al. 1979 [42]	4 HTG	48 (36–57)	IP, USA	C	Met	Yes	33–46	Liquid	Dextromaltose	45:40:15	Negative	2 wks	A, I
Turner et al. 1979 (DM2) [42]	2 HTG+ DM2	41 (40–42)	IP, USA	C	Met	Yes	33–46	Liquid	Dextromaltose	45:40:15	Negative	2 wks	A, I
Rizkalla et al. 1986 (E1) [43,44]	23 OW/OB	22 (2)	OP, France	P	Met	Yes	36	Liquid	Glucose, Galactose	25:25:50	Negative	2 wks	A, I
Rizkalla et al. 1986 (E2) [43,44]	18 OW/OB	22 (2)	OP, France	P	Met	Yes	36	Liquid	Glucose, Galactose	25:25:50	Negative	2 wks	A, I
Paganus et al. 1987 (guar) [45]	22 DM1	12.2 (8.9–15.9)	OP, Finland	C	Supp	Yes	37	Mixed	Starch	50:30:20	Neutral	3 wks	I
Paganus et al. 1987 (control) [45]	8 DM1	12.3 (10.7–14.8)	OP, Finland	C	Supp	Yes	37	Mixed	Starch	50:30:20	Neutral	3 wks	I
Grigoresco et al. 1988 [46]	8 DM2	40 (6.9)	OP, France	C	Supp	Yes	30	Liquid	Starch	50:30:20	Negative	8 wks	A, I
Blayo et al. 1990 [47]	14 DM1, 6 DM2	46.9 (13.1)	OP, France	P	Supp	Yes	20–30	Mixed	Starch, Sucrose	55:30:15	Negative	52 wks	A, I
Sunehag et al. 2002 [48]	36 H	12.4 (3.4)	IP/OP, USA	P	Met	Yes	~35.5	Mixed	Starch	60:25:15	Neutral	1 wks	A
Vaisman et al. 2006 [49]	25 DM2	65.4 (10.7)	OP, Israel	P	Supp	Yes	22.5	-	Starch	-	Neutral	12 wks	-
Aeberli et al. 2011 [50]	29 H	26.3 (6.6)	OP, Switzerland	C	Supp	Yes	40	Liquid	Glucose	-	Positive	3 wks	A, I
Heden et al. 2014 [51]	40 H+ OW/OB	17.9 (1.9)	OP, USA	C	Supp	Yes	50	Liquid	Glucose	-	Positive	2 wks	A
Lowndes et al. 2015 [52]	95 H+ OW/OB	36.3 (11.0)	OP, USA	P	Supp	Yes	45	Liquid	Glucose, Control	-	Positive	10 wks	I
Heden et al. 2015 [53]	7 OB	18 (1.1)	OP, USA	C	Supp	Yes	50	Liquid	Glucose	-	Positive	2 wks	A
**ALLULOSE**													
Hayashi et al. 2010 [11]	17 H	34 (3.7)	OP, Japan	P	Supp	Yes	15	Liquid	Glucose	-	Neutral	12 wks	I
Han et al. 2018—low dose [54]	60 OW/OB	27.2 (6.5)	OP, Korea	P	Supp	Yes	8	Liquid	Sucralose	-	Neutral	12 wks	A
Han et al. 2018—high dose [54]	61 OW/OB	26.2 (6.1)	OP, Korea	P	Supp	Yes	14	Liquid	Sucralose	-	Neutral	12 wks	A
**TAGATOSE**													
Buemann et al. 1998 [55]	8 H	26.2 (2.8)	OP, Denmark	C	Supp	Yes	30	Solid	Sucrose	-	Neutral	2 wks	A, I
Boesch et al. 2001 [56]	12 H	(21-30)	OP, Switzerland	C	Supp	No	45	Mixed	Sucrose	-	-	4 wks	-
Ensor et al. 2015 [57]	356 DM2	51.7 (10.4)	OP, India & USA	P	Supp	Yes	45	Liquid	Splenda®	-	Neutral	40 wks	A, I

HTG, hypertriglyceridemia; DM1, diabetes mellitus type 1; DM2, diabetes mellitus type 2; H, healthy; OW, overweight; OB, obese; SD, standard deviation; IP, inpatient; OP, outpatient; C, crossover; P, parallel; Met, metabolic; Supp, supplemented; A, agency; I, industry. * Met feeding control represents the provision of all meals, snacks, and study supplements (test sugars and foods) during the study. Supp feeding control represents provision of study supplements. ^†^ Doses preceded by “~” represent average doses, where fructose was administered on % energy or g/kg body-weight basis. Doses preceded by “net” represent the net difference between treatment (fructose) dose and comparator dose when treatment arms contained small amounts of the comparator, vice versa. ^‡^ Test sugar was provided in one of three forms: (1) a liquid form, where all or most of the test sugar was provided as beverages or crystalline powder to be added to beverages; (2) in a mixed form, where all or most of the test sugar was provided as beverages, solid foods, and/or crystalline fructose to be added to beverages and/or foods; or (3) a solid form, where the test sugar was administered in the form of a solid food. Comparator refers to the reference carbohydrate or control group (e.g., starch, sucrose, glucose). ** Values are for the ratio of carbohydrate:fat:protein. *** Agency funding represents funding from government, university, or not-for-profit health agency sources.

## Supplementary Materials

The following are available online at http://www.mdpi.com/2072-6643/10/11/1805/s1, Figure S1: Risk of bias summary of controlled feeding trials assessing the effect of small doses of fructose (top), allulose (middle) and tagatose (bottom) on markers of glycemic control. Figure S2: Forest plot of the effect of small doses (≤50 g/day) of fructose on HbA_1c_. Figure S3: Forest plot of the effect of small doses (≤50 g/day) of fructose on fasting glucose. Figure S4: Forest plot of the effect of small doses (≤50 g/day) fructose on fasting insulin. Figure S5: Forest plot of the effect of small doses (≤50 g/day) of allulose on HbA_1c_. Figure S6: Forest plot of the effect of small doses (≤50g/day) of allulose on fasting glucose. Figure S7: Forest plot of the effect of small doses (≤50 g/day) of allulose on fasting insulin. Figure S8: Forest plot of the effect of small doses (≤50 g/day) of tagatose on HbA_1c_. Figure S9: Forest plot of the effect of small doses (≤50 g/day) of tagatose on fasting glucose. Figure S10: Forest plot of the effect of small doses (≤50 g/day) of tagatose on fasting insulin. Figure S11: Forest plot of subgroup analyses investigating the effect of small doses of fructose on fasting glucose. Figure S12: Forest plot of subgroup analyses investigating the effect of small doses of fructose on fasting glucose. Figure S13: Forest plot of subgroup analyses investigating the effect of small doses of fructose on fasting insulin. Figure S14: Forest plot of subgroup analyses investigating the effect of small doses of fructose on fasting insulin. Figure S15: Publication bias funnel plots for the effect of small doses (≤50 g/d) of fructose on fasting glucose (top) and fasting insulin (bottom). Table S1: Search strategy for studies assessing the effect of fructose and its epimers (allulose, tagatose and sorbose) on markers of long-term glycemic control. Table S2: Select sensitivity analyses in which the systematic removal of an individual trial altered the effect estimate or the evidence for heterogeneity. Table S3: Sensitivity analyses using correlation coefficients of 0.25 and 0.75 for crossover trials. Table S4: GRADE assessment.
