# Supplementary material for: The Effect of Small Doses of Fructose and Its Epimers on Glycemic Control: A Systematic Review and Meta-Analysis of Controlled Feeding Trials
Source: Nutrients. 2018 Nov 20;10(11):1805. doi: 10.3390/nu10111805 (PMC6266436; doi:10.3390/nu10111805)
Supplement: Supplementary file 1 [file nutrients-10-01805-s001.pdf]

Table S1. Search strategy for studies assessing the effect of fructose and its epimers (allulose, tagatose and sorbose) on markers of long-term glycemic control

Database	Search Period	Search Terms
MEDLINE	1946 to April 18, 2018	1. exp Fructose/<br>2. psicose.mp.<br>3. allulose.mp.<br>4. tagatose.mp.<br>5. sorbose.mp.<br>6. 1 or 2 or 3 or 4 or 5<br>7. exp Glucose/<br>8. glycaemic.mp.<br>9. glycemic.mp.<br>10. glycaemia.mp.<br>11. glycemia.mp.<br>12. exp Insulin/<br>13. exp Glucose Tolerance Test/<br>14. OGTT.mp.<br>15. exp Hemoglobin A, Glycosylated/<br>16. HbA1c.mp.<br>17. 7 or 8 or 9 or 10 or 11 or 12 or 13 or 14 or 15 or 16<br>18. 6 and 17<br>19. limit 18 to animals<br>20. 18 not 19<br>21. clinical trial.mp.<br>22. clinical trial.pt.<br>23. random:.mp.<br>24. tu.xs.<br>25. 21 or 22 or 23 or 24<br>26. 20 and 25
EMBASE	1946 to April 18, 2018	1. exp fructose/<br>2. psicose.mp.<br>3. allulose.mp.<br>4. tagatose.mp.<br>5. sorbose.mp.<br>6. 1 or 2 or 3 or 4 or 5<br>7. exp glucose/<br>8. glycaemic.mp.<br>9. glycemic.mp.<br>10. glycaemia.mp.<br>11. glycemia.mp.<br>12. exp insulin/<br>13. exp oral glucose tolerance test/<br>14. OGTT.mp.<br>15. exp hemoglobin A1c/<br>16. HbA1c.mp.<br>17. 7 or 8 or 9 or 10 or 11 or 12 or 13 or 14 or 15 or 16<br>18. 6 and 17<br>19. limit 18 to animals<br>20. 18 not 19<br>21. random:.tw.<br>22. clinical trial:.mp.<br>23. exp health care quality/

		24. 21 or 22 or 23<br>25. 20 and 24
Cochrane Central Register of Controlled Trials	1946 to April 18, 2018	1. Fructose/<br>2. psicose.mp.<br>3. tagatose.mp.<br>4. 1 or 2 or 3<br>5. Glucose/<br>6. glycaemic.mp.<br>7. glycemic.mp.<br>8. glycaemia.mp.<br>9. glycemia.mp.<br>10. Insulin/<br>11. exp Glucose Tolerance Test/<br>12. OGTT.mp.<br>13. exp Hemoglobin A, Glycosylated/<br>14. HbA1c.mp.<br>15. 5 or 6 or 7 or 8 or 9 or 10 or 11 or 12 or 13 or 14<br>16. 4 and 15

Table S2. Select sensitivity analyses in which the systematic removal of an individual trial altered the effect estimate or the evidence for heterogeneity

Outcome	Removal of	MD [95% CI], P-value<br>I^2, P-value
**ALLULOSE**		
**Fasting glucose, mmol/l**	Hayashi et al.	-0.28 [-0.52, -0.04], p=0.02<br>I^2=0%, P_Q=0.57
**TAGATOSE**		
**Fasting glucose, mmol/l**	Ensor et al.	-0.12 [-0.61, 0.37], p=0.63<br>I^2=NA P_Q=NA

Table S3. Sensitivity analyses using correlation coefficients of 0.25 and 0.75 for crossover trials

Outcome<br>(No. crossover trials/total trials)	MD [95% CI], P-value<br>I^2, P-value	
	**Correlation coefficient of 0.25**	**Correlation coefficient of 0.75**
**FRUCTOSE**		
**HbA_{1c}, %**<br>**(3/7)**	-0.40 [-0.67, -0.13], p=0.003<br>I^2 = 0%, P_Q=0.57	-0.34 [-0.62, -0.06], p=0.02<br>I^2 = 33%, P_Q=0.18
**Fasting glucose, mmol/L**<br>**(7/12)**	-0.14 [-0.25, -0.03], p=0.01<br>I^2 = 30%, P_Q=0.16	-0.12 [-0.27, 0.02], p=0.10<br>I^2 = 48%, P_Q=0.04
**Fasting insulin, pmol/L**<br>**(6/10)**	3.78 [-3.46, 11.02], p=0.31<br>I^2 = 0%, P_Q=0.64	-1.50 [-8.83, 5.82], p=0.69<br>I^2 = 13%, P_Q=0.33
**TAGATOSE**		
**Fasting glucose, mmol/L**<br>**(1/2)**	-0.33 [-0.60, -0.05], p=0.02<br>I^2 = 0%, P_Q=0.46	-0.27 [-0.50, -0.03], p=0.03<br>I^2 = 14%, P_Q=0.28
**Fasting insulin, pmol/L**<br>**(2/3)**	-1.12 [-6.86, 4.63], p=0.70<br>I^2 = 36%, P_Q=0.21	-2.42 [-7.00, 2.15], p=0.30<br>I^2 = 70%, P_Q=0.04

Table S4. GRADE assessment

Grading of Recommendations, Assessment, Development and Evaluation (GRADE)							Quality
No. of studies	Study design	Risk of bias	Inconsistency	Indirectness	Imprecision	Other considerations	
**HbA_{1c} in FRUCTOSE trials**							
7	randomized trials	not serious	not serious	serious[1]	serious[2]	none[15]	⊕⊕◯◯ LOW
**Fasting glucose in FRUCTOSE trials**							
12	randomized trials	not serious	not serious	serious[3]	serious[4]	none	⊕⊕◯◯ LOW
**Fasting insulin in FRUCTOSE trials**							
10	randomized trials	not serious	not serious	serious[5]	serious[6]	none	⊕⊕◯◯ LOW
**HbA_{1c} in ALLULOSE trials**							
2	randomized trials	not serious	not serious	serious[7]	serious[8]	none[15]	⊕⊕◯◯ LOW
**Fasting glucose in ALLULOSE trials**							
2	randomized trials	not serious	not serious	serious[7]	serious[9]	none[15]	⊕⊕◯◯ LOW
**Fasting insulin in ALLULOSE trials**							
2	randomized trials	not serious	not serious	serious[7]	serious[10]	none[15]	⊕⊕◯◯ LOW
**HbA_{1c} in TAGATOSE trial**							
1	randomized trials	not serious	not serious	not serious[11]	serious[12]	none[15]	⊕⊕⊕◯ MODERATE
**Fasting glucose in TAGATOSE trials**							
2	randomized and non-randomized trials	not serious	not serious	not serious[11]	serious[13]	none[15]	⊕⊕⊕◯ MODERATE
**Fasting insulin in TAGATOSE trials**							
3	randomized and non-randomized trials	not serious	serious	not serious[11]	serious[14]	none[15]	⊕⊕⊕◯ MODERATE

[1] Serious indirectness for the effect of small doses of fructose on HbA_{1c}, as the median follow-up duration was relatively short (2.5 weeks) Only 3/7 trials had a follow-up duration of ≥8-weeks.

[2]Serious imprecision for the effect of small doses of fructose on HbA_{1c}, as the 95% CIs (-0.64% to -0.13%) overlap the minimally important difference for clinical benefit (0.3%)

[3]Serious indirectness for the effect of small doses of fructose on fasting glucose, as the median follow-up duration was relatively short (2 weeks).

[4]Serious imprecision for the effect of small doses of fructose on fasting glucose, as the 95% CIs (-0.24 mmol/l to -0.03 mmol/l) overlap the minimally important difference for clinical benefit (0.5 mmol/l)

[5]Serious indirectness for the effect of small doses of fructose on fasting insulin, as the median follow-up duration was relatively short (2 weeks).

[6]Serious imprecision for the effect of small doses of fructose on fasting insulin, as the 95% CIs (-4.19 pmol/l to 9.62 pmol/l) overlap the minimally important difference for clinical benefit (5 pmol/l)

[7]Serious indirectness for the effect of small doses of allulose on HbA_{1c}, fasting glucose and fasting insulin, as the study setting was limited to Asia (Japan and Korea) which may affect the generalizability of the results

[8]Serious imprecision for the effect of small doses of allulose on HbA_{1c}, as the 95% CIs (-0.03% to 0.07%) overlap the minimally important difference for clinical benefit (0.3%)

[9]Serious imprecision for the effect of small doses of allulose on fasting glucose, as the 95% CIs (-0.35 mmol/l to 0.00 mmol/l) overlap the minimally important difference for clinical benefit (0.5 mmol/l)

[10]Serious imprecision for the effect of small doses of allulose on fasting insulin, as the 95% CIs (-2.17 pmol/l to 0.79 pmol/l) overlap the minimally important difference for clinical benefit (5 pmol/l)

[11]No serious indirectness of the effect of small doses of tagatose on HbA_{1c}, fasting glucose, and fasting insulin, as 356 – 378 participants were included in the analysis even though 1 – 3 trials were available. Trials were of sufficient length and assessed the effect of small doses of tagatose in a large population of interest (i.e. type 2 diabetes). The one multi-center trial (USA & India) studying 356 participants with type 2 diabetes had a follow-up duration of 40 weeks.

[12]Serious imprecision for the effect of small doses of tagatose on HbA_{1c}, as the 95% CIs (-0.34% to -0.06%) overlap the minimally important difference for clinical benefit (0.3%)

[13]Serious imprecision for the effect of small doses of tagatose on fasting glucose, as the 95% CIs (-0.57 mmol/l to 0.04 mmol/l) overlap the minimally important difference for clinical benefit (0.5 mmol/l)

[14]Serious imprecision for the effect of small doses of tagatose on fasting insulin, as the 95% CIs (-6.95 pmol/l to 3.77 pmol/l) overlap the minimally important difference for clinical benefit (5 pmol/l)

[15]Not able to assess publication bias as <10 trials were available

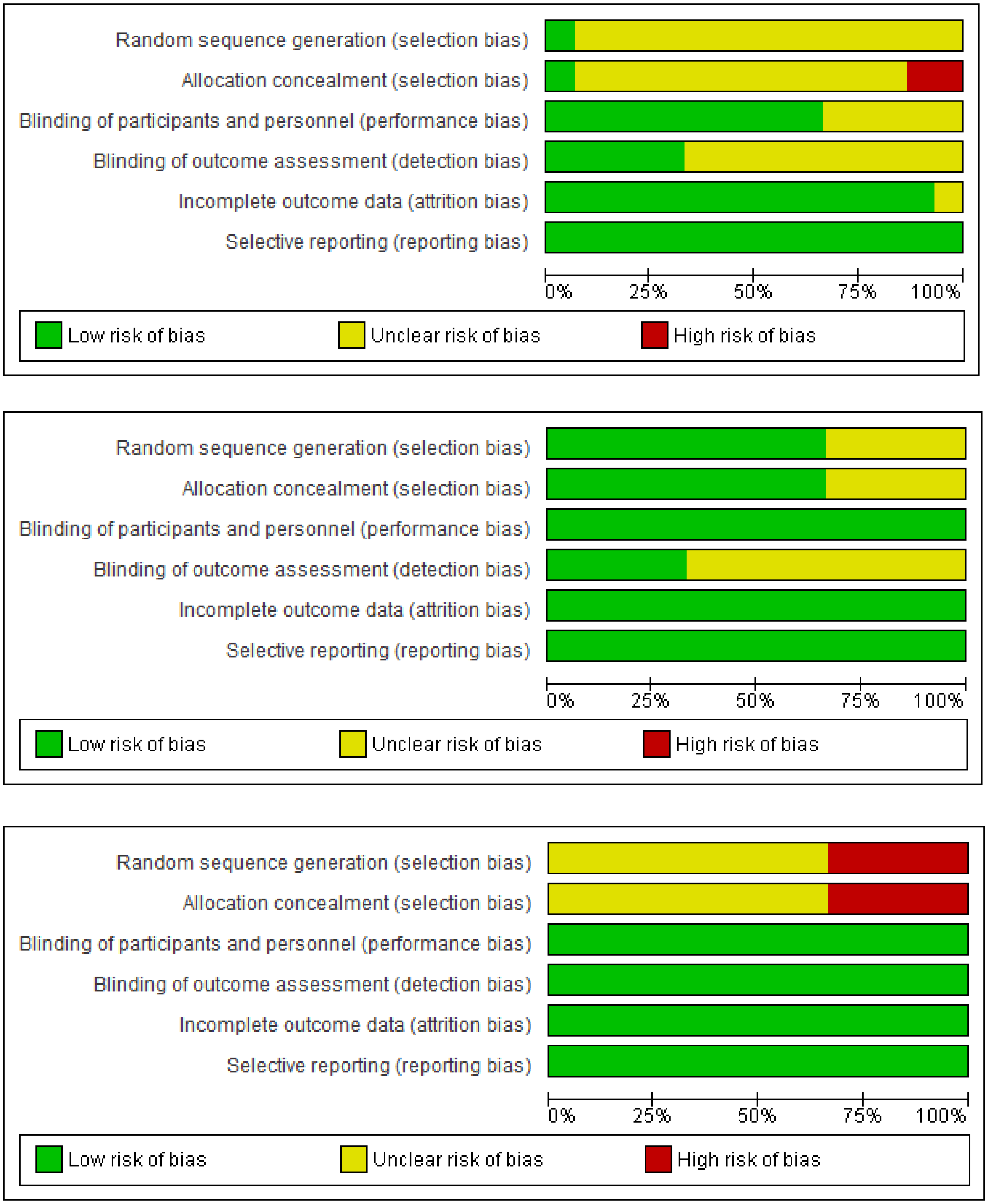

Figure S1. Risk of bias summary of controlled feeding trials assessing the effect of small doses of fructose (top), allulose (middle) and tagatose (bottom) on markers of glycemic control. Colored bars represent the proportion of studies assessed as low (green), unclear (yellow) and high (red) risk of bias for the 6 domains of bias above according to criteria set by the Cochrane Risk of Bias tool.

Study or Subgroup	Year	Intervention, N	Control, N	Weight	Mean Difference [95% CI] in HbA_{1c}, %
**Overweight/Obese**					
Rizkalla et al. (Expt 1) [43,44]	1986	8	8	13.7%	-0.50 [-1.19, 0.19]
Rizkalla et al. (Expt 2) [43,44]	1986	6	6	13.0%	-0.60 [-1.31, 0.11]
**Subtotal**					**-0.55 [-1.04, -0.06]**
Heterogeneity: $Tau^2 = 0.00$; $Chi^2 = 0.04$, df = 1 (P = 0.84); $I^2 = 0\%$					
Test for overall effect: Z = 2.19 (P = 0.03)					
**Type 1 Diabetes**					
Paganus et al. (control) [45]	1987	8	8	17.5%	-0.30 [-0.91, 0.31]
Paganus et al. (guar) [45]	1987	22	22	11.6%	-0.50 [-1.24, 0.24]
**Subtotal**					**-0.38 [-0.85, 0.09]**
Heterogeneity: $Tau^2 = 0.00$; $Chi^2 = 0.17$, df = 1 (P = 0.68); $I^2 = 0\%$					
Test for overall effect: Z = 1.58 (P = 0.11)					
**Type 2 Diabetes**					
Grigoresco et al. [46]	1988	8	8	7.0%	0.50 [-0.46, 1.46]
Vaisman et al. [49]	2006	12	13	24.9%	-0.22 [-0.73, 0.29]
**Subtotal**					**0.02 [-0.65, 0.69]**
Heterogeneity: $Tau^2 = 0.11$; $Chi^2 = 1.68$, df = 1 (P = 0.19); $I^2 = 41\%$					
Test for overall effect: Z = 0.06 (P = 0.95)					
**Mixed (T1D and T2D)**					
Blayo et al. [47]	1990	6	8	12.3%	-0.85 [-1.58, -0.12]
**Subtotal**					**-0.85 [-1.58, -0.12]**
Heterogeneity: Not applicable					
Test for overall effect: Z = 2.30 (P = 0.02)					
**Total**					**-0.38 [-0.64, -0.13]**
Heterogeneity: $Tau^2 = 0.00$; $Chi^2 = 5.87$, df = 6 (P = 0.44); $I^2 = 0\%$					
Test for overall effect: Z = 2.94 (P = 0.003)					
Test for subgroup differences: $Chi^2 = 3.31$, df = 3 (P = 0.35), $I^2 = 9.5\%$					

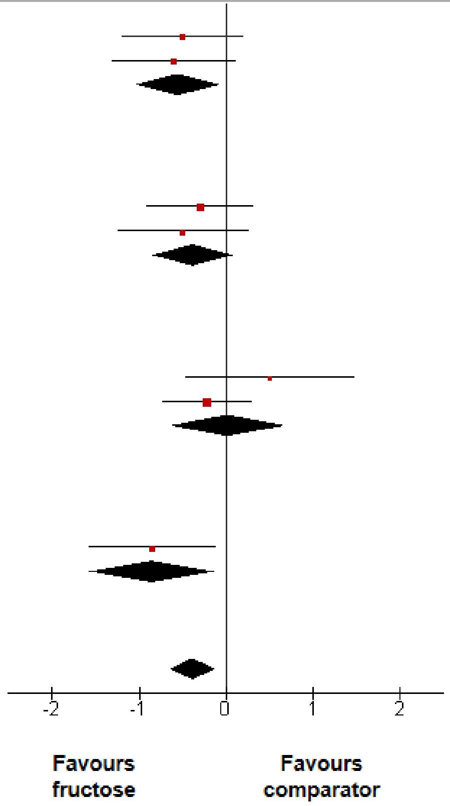

Figure S2. Forest plot of the effect of small doses (≤50g/day) of fructose on HbA_{1c}. Pooled effect estimates for the overall effect is represented by the diamond. Data are expressed as mean differences (MDs) with 95% confidence intervals (CIs), using the generic inverse variance method with random effects models. Paired analyses were applied to all crossover trials. Values are between-treatment end differences for six of the seven trials (Rizkalla *et al.* (Expt 1) [43], Paganus *et al.* (control) [45], Paganus *et al.* (guar) [45], Grigoresco *et al.* [46], Vaisman *et al.* [49], Blayo *et al.* [47]), as change-from-baseline data were not available. Inter-study heterogeneity was tested by the Cochran Q-statistic at a significance level of p<0.10 and quantified by I^2, levels ≤ 50% represent moderate heterogeneity, ≥ 50% representing substantial heterogeneity, and ≥ 75% representing considerable heterogeneity.

Study or Subgroup	Year	Intervention, N	Control, N	Weight	Mean Difference [95% CI] in fasting glucose, mmol/l
**Healthy**					
Sunehag et al. [48]	2002	24	12	11.6%	-0.30 [-0.54, -0.06]
Aeberli et al. [50]	2011	29	29	20.8%	-0.11 [-0.23, 0.01]
**Subtotal**					**-0.18 [-0.35, 0.00]**
Heterogeneity: Tau^2 = 0.01; Chi^2 = 2.01, df = 1 (P = 0.16); I^2 = 50%					
Test for overall effect: Z = 1.95 (P = 0.05)					
**Mixed (Lean and Overweight/Obese)**					
Heden et al. [51]	2014	40	40	14.2%	-0.11 [-0.31, 0.09]
Lowndes et al. [52]	2015	30	34	11.6%	-0.10 [-0.34, 0.14]
**Subtotal**					**-0.11 [-0.26, 0.04]**
Heterogeneity: Tau^2 = 0.00; Chi^2 = 0.00, df = 1 (P = 0.95); I^2 = 0%					
Test for overall effect: Z = 1.38 (P = 0.17)					
**Overweight/obese**					
Rizkalla et al. (Expt 1) [43,44]	1986	8	8	12.8%	-0.11 [-0.33, 0.11]
Rizkalla et al. (Expt 2) [43,44]	1986	6	6	5.7%	0.06 [-0.33, 0.45]
Heden et al. (- exercise) [53]	2015	7	7	4.9%	-0.05 [-0.48, 0.38]
Heden et al. (+ exercise) [53]	2015	7	7	4.9%	0.15 [-0.28, 0.58]
**Subtotal**					**-0.04 [-0.20, 0.12]**
Heterogeneity: Tau^2 = 0.00; Chi^2 = 1.40, df = 3 (P = 0.71); I^2 = 0%					
Test for overall effect: Z = 0.45 (P = 0.65)					
**Hypertriglyceridemia**					
Turner et al. [42]	1979	4	4	6.7%	-0.18 [-0.53, 0.17]
**Subtotal**					**-0.18 [-0.53, 0.17]**
Heterogeneity: Not applicable					
Test for overall effect: Z = 1.00 (P = 0.32)					
**Type 2 Diabetes**					
Turner et al. (DM2) [42]	1979	2	2	1.7%	0.57 [-0.21, 1.35]
Grigoresco et al. [46]	1988	8	8	0.3%	-0.40 [-2.26, 1.46]
**Subtotal**					**0.42 [-0.30, 1.15]**
Heterogeneity: Tau^2 = 0.00; Chi^2 = 0.89, df = 1 (P = 0.35); I^2 = 0%					
Test for overall effect: Z = 1.15 (P = 0.25)					
**Mixed (T1D and T2D)**					
Blayo et al. [47]	1990	6	8	4.9%	-0.78 [-1.21, -0.35]
**Subtotal**					**-0.78 [-1.21, -0.35]**
Heterogeneity: Not applicable					
Test for overall effect: Z = 3.55 (P = 0.0004)					
**Total**					**-0.13 [-0.24, -0.03]**
Heterogeneity: Tau^2 = 0.01; Chi^2 = 16.86, df = 11 (P = 0.11); I^2 = 35%					
Test for overall effect: Z = 2.48 (P = 0.01)					
Test for subgroup differences: Chi^2 = 12.71, df = 5 (P = 0.03), I^2 = 60.7%					

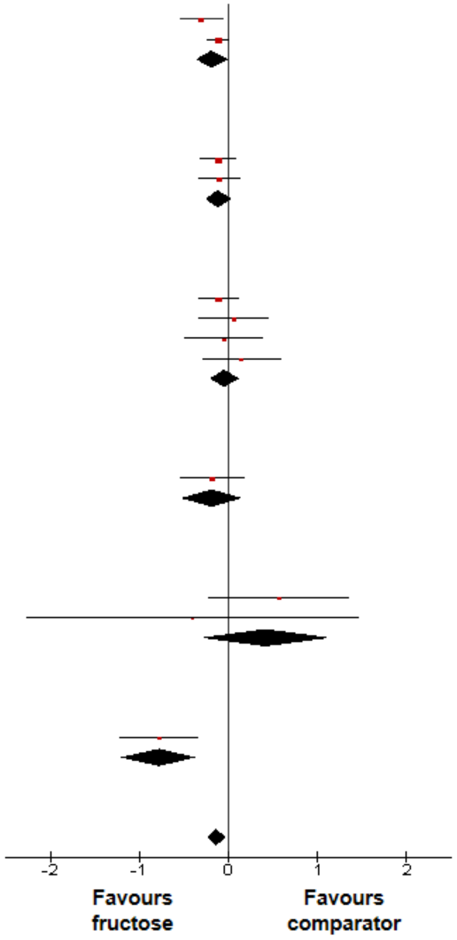

Figure S3. Forest plot of the effect of small doses (≤50g/day) of fructose on fasting glucose. Pooled effect estimates for the overall effect is represented by the diamond. Data are expressed as mean differences (MDs) with 95% confidence intervals (CIs), using the generic inverse variance method with random effects models. Paired analyses were applied to all crossover trials. Values are between-treatment end differences for all trials, as change-from-baseline data were not available. Inter-study heterogeneity was tested by the Cochran Q-statistic at a significance level of p<0.10 and quantified by I^2, levels ≤ 50% represent moderate heterogeneity, ≥ 50% representing substantial heterogeneity, and ≥ 75% representing considerable heterogeneity.

Study or Subgroup	Year	Intervention, N	Control, N	Weight	Mean Difference [95% CI] in fasting insulin, pmol/l
**Healthy**					
Sunehag et al. [48]	2002	24	12	33.9%	2.08 [-9.78, 13.94]
**Subtotal**					**2.08 [-9.78, 13.94]**
Heterogeneity: Not applicable					
Test for overall effect: Z = 0.34 (P = 0.73)					
**Mixed (Lean and Overweight/Obese)**					
Heden et al. [51]	2014	40	40	18.5%	-5.00 [-21.05, 11.05]
Lowndes et al. [52]	2015	30	34	9.8%	22.30 [0.21, 44.39]
**Subtotal**					**7.55 [-19.11, 34.22]**
Heterogeneity: Tau² = 275.60; Chi² = 3.84, df = 1 (P = 0.05); I² = 74%					
Test for overall effect: Z = 0.56 (P = 0.58)					
**Overweight/obese**					
Rizkalla et al. (Expt 1) [43,44]	1986	8	8	11.0%	6.95 [-13.85, 27.75]
Rizkalla et al. (Expt 2) [43,44]	1986	6	6	17.0%	7.64 [-9.12, 24.40]
Heden et al. (- exercise) [53]	2015	7	7	2.5%	-24.45 [-68.06, 19.16]
Heden et al. (+ exercise) [53]	2015	7	7	2.5%	14.85 [-28.76, 58.46]
**Subtotal**					**5.52 [-6.50, 17.54]**
Heterogeneity: Tau² = 0.00; Chi² = 2.07, df = 3 (P = 0.56); I² = 0%					
Test for overall effect: Z = 0.90 (P = 0.37)					
**Hypertriglyceridemia**					
Turner et al. [42]	1979	4	4	3.5%	-24.70 [-61.49, 12.09]
**Subtotal**					**-24.70 [-61.49, 12.09]**
Heterogeneity: Not applicable					
Test for overall effect: Z = 1.32 (P = 0.19)					
**Type 2 Diabetes**					
Turner et al. (DM2) [42]	1979	2	2	0.7%	-24.61 [-105.26, 56.04]
Grigoresco et al.	1988	8	8	0.6%	-2.08 [-91.53, 87.37]
**Subtotal**					**-14.51 [-74.41, 45.39]**
Heterogeneity: Tau² = 0.00; Chi² = 0.13, df = 1 (P = 0.71); I² = 0%					
Test for overall effect: Z = 0.47 (P = 0.64)					
**Total**					**2.72 [-4.19, 9.62]**
Heterogeneity: Tau² = 0.00; Chi² = 8.78, df = 9 (P = 0.46); I² = 0%					
Test for overall effect: Z = 0.77 (P = 0.44)					
Test for subgroup differences: Chi² = 2.80, df = 4 (P = 0.59), I² = 0%					

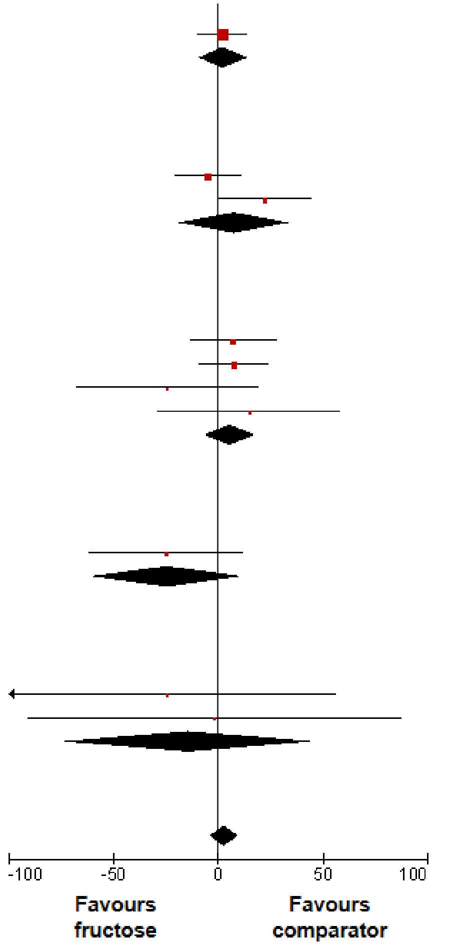

Figure S4. Forest plot of the effect of small doses (≤50g/day) fructose on fasting insulin. Pooled effect estimates for the overall effect is represented by the diamond. Data are expressed as mean differences (MDs) with 95% confidence intervals (CIs), using the generic inverse variance method with random effects models. Paired analyses were applied to all crossover trials. Values are between-treatment end differences for all trials, as change-from-baseline data were not available. Inter-study heterogeneity was tested by the Cochran Q-statistic at a significance level of p<0.10 and quantified by I^2, levels ≤50% represent moderate heterogeneity, ≥50% representing substantial heterogeneity, and ≥75% representing considerable heterogeneity.

Study or Subgroup	Year	Intervention, N	Control, N	Weight	Mean Difference [95% CI] in HbA_{1c}, %
**Normal**					
Hayashi et al. [11]	2010	17	17	14.0%	0.10 [-0.04, 0.24]
**Subtotal**					**0.10 [-0.04, 0.24]**
Heterogeneity: Not applicable					
Test for overall effect: Z = 1.43 (P = 0.15)					
**Overweight/obese**					
Han et al. (high dose) [54]	2018	40	20	43.0%	0.02 [-0.06, 0.10]
Han et al. (low dose) [54]	2018	41	20	43.0%	-0.01 [-0.09, 0.07]
**Subtotal**					**0.01 [-0.05, 0.06]**
Heterogeneity: $Chi^2 = 0.28$, df = 1 (P = 0.60); $I^2 = 0\%$					
Test for overall effect: Z = 0.18 (P = 0.86)					
**Total**					**0.02 [-0.03, 0.07]**
Heterogeneity: $Chi^2 = 1.86$, df = 2 (P = 0.39); $I^2 = 0\%$					
Test for overall effect: Z = 0.70 (P = 0.48)					
Test for subgroup differences: $Chi^2 = 1.58$, df = 1 (P = 0.21), $I^2 = 36.8\%$					

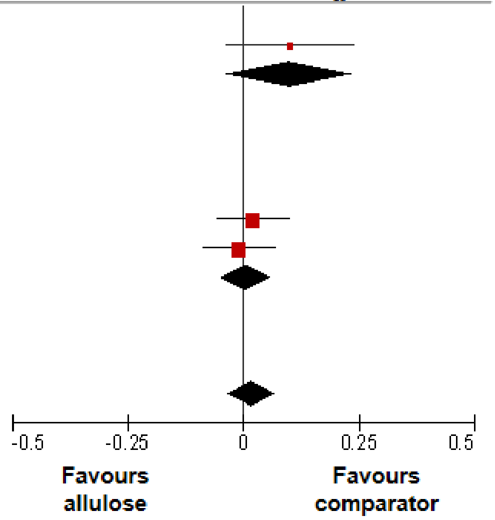

Figure S5. Forest plot of the effect of small doses (≤50g/day) of allulose on HbA_{1c}. Pooled effect estimate for the overall effect is represented by the diamond. Data are expressed as mean differences (MDs) with 95% confidence intervals (CIs), using the generic inverse variance method with fixed effects models. Paired analyses were applied to all crossover trials. Values are between-treatment end differences for Hayashi *et al.* [11], as change-from-baseline data were not available. Inter-study heterogeneity was tested by the Cochran Q-statistic at a significance level of p<0.10 and quantified by I^2, levels ≤50% represent moderate heterogeneity, ≥50% representing substantial heterogeneity, and ≥75% representing considerable heterogeneity

Study or Subgroup	Year	Intervention, N	Control, N	Weight	Mean Difference [95% CI] in fasting glucose, mmol/l
**Normal**					
Hayashi et al. [11]	2010	17	17	47.0%	-0.06 [-0.31, 0.19]
**Subtotal**					**-0.06 [-0.31, 0.19]**
Heterogeneity: Not applicable					
Test for overall effect: Z = 0.46 (P = 0.64)					
**Overweight/obese**					
Han et al. (high dose) [54]	2018	41	20	22.0%	-0.20 [-0.57, 0.17]
Han et al. (low dose) [54]	2018	40	20	31.0%	-0.34 [-0.65, -0.03]
**Subtotal**					-0.28 [-0.52, -0.04]
Heterogeneity: Chi² = 0.32, df = 1 (P = 0.57); I² = 0%					
Test for overall effect: Z = 2.30 (P = 0.02)					
**Total**					**-0.18 [-0.35, -0.00]**
Heterogeneity: Chi² = 1.86, df = 2 (P = 0.39); I² = 0%					
Test for overall effect: Z = 1.99 (P = 0.05)					
Test for subgroup differences: Chi² = 1.54, df = 1 (P = 0.21), I² = 35.3%					

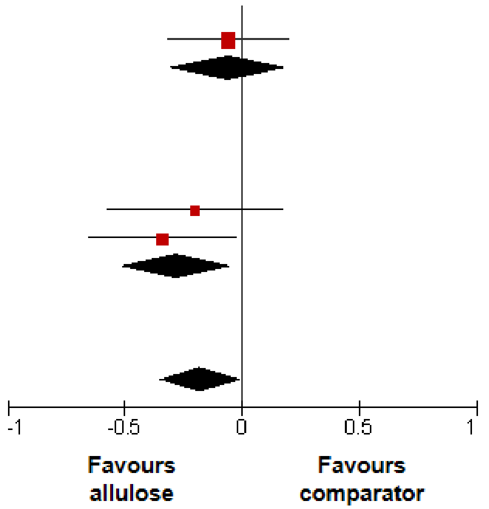

Figure S6. Forest plot of the effect of small doses (≤50g/day) of allulose on fasting glucose. Pooled effect estimate for the overall effect is represented by the diamond. Data are expressed as mean differences (MDs) with 95% confidence intervals (CIs), using the generic inverse variance method with fixed effects models. Paired analyses were applied to all crossover trials. Values are between-treatment end differences for Hayashi *et al.* [11], as change-from-baseline data were not available. Inter-study heterogeneity was tested by the Cochran Q-statistic at a significance level of p<0.10 and quantified by I^2, levels ≤50% represent moderate heterogeneity, ≥50% representing substantial heterogeneity, and ≥75% representing considerable heterogeneity

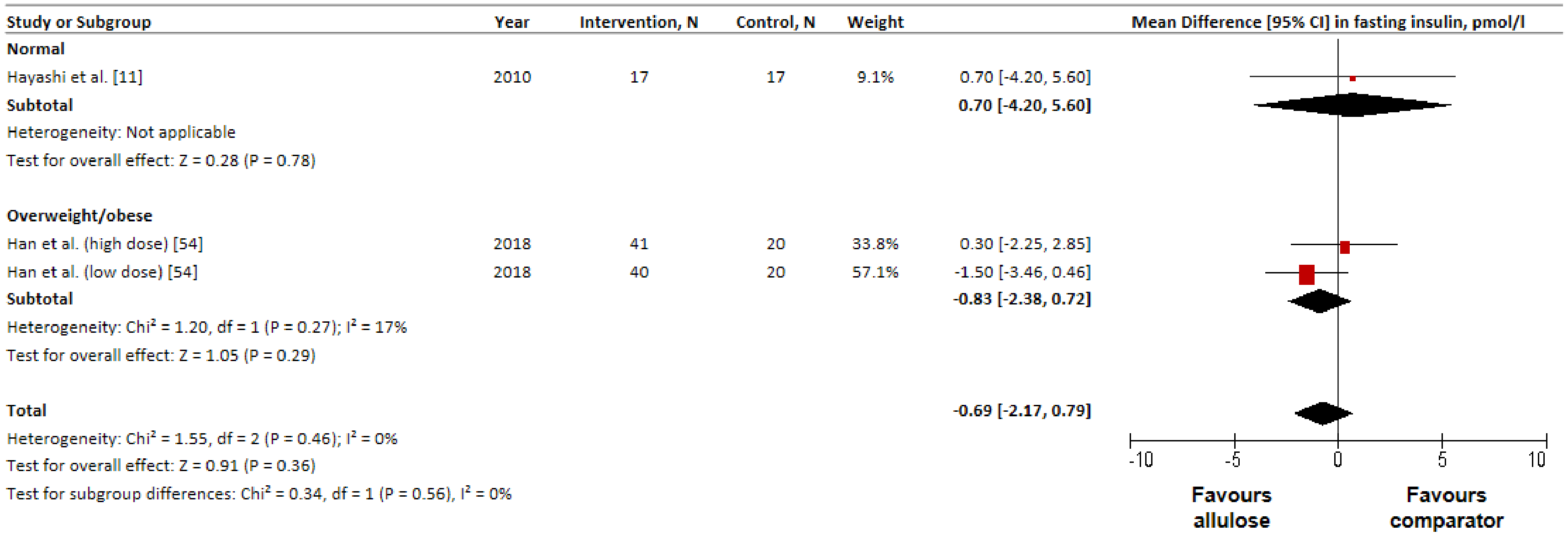

Figure S7. Forest plot of the effect of small doses (≤50g/day) of allulose on fasting insulin. Pooled effect estimate for the overall effect is represented by the diamond. Data are expressed as mean differences (MDs) with 95% confidence intervals (CIs), using the generic inverse variance method with fixed effects models. Paired analyses were applied to all crossover trials. Values are between-treatment end differences for Hayashi *et al.* [11], as change-from-baseline data were not available. Inter-study heterogeneity was tested by the Cochran Q-statistic at a significance level of $p<0.10$ and quantified by I^2, levels ≤50% represent moderate heterogeneity, ≥50% representing substantial heterogeneity, and ≥75% representing considerable heterogeneity

Study or Subgroup	Year	Intervention, N	Control, N	Weight	Mean Difference [95% CI] in HbA1c, %
**Type 2 Diabetes**					
Ensor et al. [57]	2014	172	184	100.0%	-0.20 [-0.34, -0.06]
**Subtotal**					**-0.20 [-0.34, -0.06]**
Heterogeneity: Not applicable					
Test for overall effect: Z = 2.86 (P = 0.004)					
**Total**					**-0.20 [-0.34, -0.06]**
Heterogeneity: Not applicable					
Test for overall effect: Z = 2.86 (P = 0.004)					
Test for subgroup differences: Not applicable					

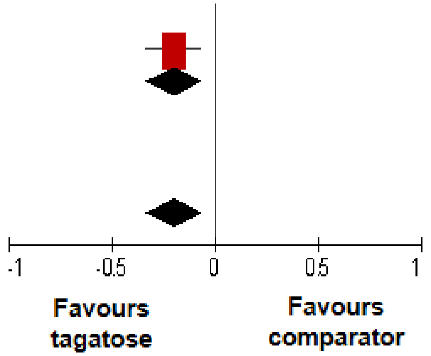

Figure S8. Forest plot of the effect of small doses (≤50g/day) of tagatose on HbA_{1c}. Pooled effect estimate for the overall effect is represented by the diamond. Data are expressed as mean differences (MDs) with 95% confidence intervals (CIs), using the generic inverse variance method with fixed effects models. Paired analyses were applied to all crossover trials. Values are between-treatment end differences, as change-from-baseline data were not available. Inter-study heterogeneity was tested by the Cochran Q-statistic at a significance level of p<0.10 and quantified by I^2, levels ≤50% represent moderate heterogeneity, ≥50% representing substantial heterogeneity, and ≥75% representing considerable heterogeneity

Study or Subgroup	Year	Intervention, N	Control, N	Weight	Mean Difference [95% CI] in fasting glucose, mmol/l
**Healthy**					
Boesch et al. [56]	2011	12	12	29.1%	-0.12 [-0.61, 0.37]
**Subtotal**					**-0.12 [-0.61, 0.37]**
Heterogeneity: Not applicable					
Test for overall effect: Z = 0.48 (P = 0.63)					
**Type 2 Diabetes**					
Ensor et al. [57]	2014	172	184	70.9%	-0.38 [-0.69, -0.07]
**Subtotal**					**-0.38 [-0.69, -0.07]**
Heterogeneity: Not applicable					
Test for overall effect: Z = 2.38 (P = 0.02)					
**Total**					**-0.30 [-0.57, -0.04]**
Heterogeneity: $Chi^2 = 0.77$, df = 1 (P = 0.38); $I^2 = 0\%$					
Test for overall effect: Z = 2.26 (P = 0.02)					
Test for subgroup differences: $Chi^2 = 0.77$, df = 1 (P = 0.38), $I^2 = 0\%$					

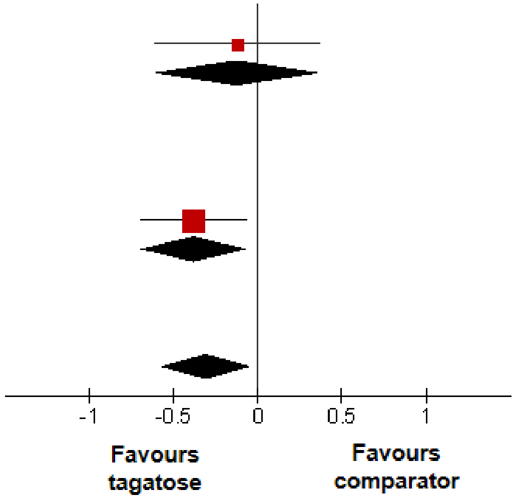

Figure S9. Forest plot of the effect of small doses (≤50g/day) of tagatose on fasting glucose. Pooled effect estimate for the overall effect is represented by the diamond. Data are expressed as mean differences (MDs) with 95% confidence intervals (CIs), using the generic inverse variance method with fixed effects models. Paired analyses were applied to all crossover trials. Values are between-treatment end differences, as change-from-baseline data were not available. Inter-study heterogeneity was tested by the Cochran Q-statistic at a significance level of p<0.10 and quantified by I^2, levels ≤50% represent moderate heterogeneity, ≥50% representing substantial heterogeneity, and ≥75% representing considerable heterogeneity

Study or Subgroup	Year	Intervention, N	Control, N	Weight	Mean Difference [95% CI] in fasting insulin, pmol/l
**Healthy**					
Buemann et al. [55]	1998	8	8	40.5%	-4.10 [-12.53, 4.33]
Boesch et al. [56]	2011	12	12	0.4%	-80.60 [-163.31, 2.11]
**Subtotal**					**-4.89 [-13.27, 3.50]**
Heterogeneity: Chi^2 = 3.25, df = 1 (P = 0.07); I^2 = 69%					
Test for overall effect: Z = 1.14 (P = 0.25)					
**Type 2 Diabetes**					
Ensor et al. [57]	2014	172	184	59.1%	0.69 [-6.29, 7.67]
**Subtotal**					**0.69 [-6.29, 7.67]**
Heterogeneity: Not applicable					
Test for overall effect: Z = 0.19 (P = 0.85)					
**Total**					**-1.59 [-6.95, 3.77]**
Heterogeneity: Chi^2 = 4.26, df = 2 (P = 0.12); I^2 = 53%					
Test for overall effect: Z = 0.58 (P = 0.56)					
Test for subgroup differences: Chi^2 = 1.00, df = 1 (P = 0.32), I^2 = 0.4%					

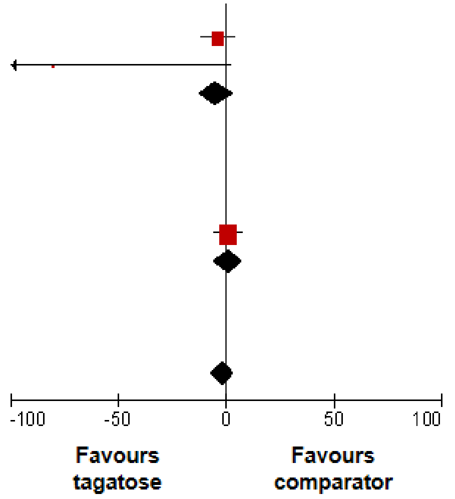

Figure S10. Forest plot of the effect of small doses (≤50g/day) of tagatose on fasting insulin. Pooled effect estimate for the overall effect is represented by the diamond. Data are expressed as mean differences (MDs) with 95% confidence intervals (CIs), using the generic inverse variance method with fixed effects models. Paired analyses were applied to all crossover trials. Values are between-treatment end differences for two of the three trials (Buemann *et al.* [55], Boesch *et al.* [56]), as change-from-baseline data were not available. Inter-study heterogeneity was tested by the Cochran Q-statistic at a significance level of p<0.10 and quantified by I^2, levels ≤50% represent moderate heterogeneity, ≥50% representing substantial heterogeneity, and ≥75% representing considerable heterogeneity

Subgroup	Level	Trials	N	Mean difference (95% CI) in fasting glucose (mmol/L): Within subgroups	Mean difference (95% CI) in fasting glucose (mmol/L): Between subgroups	Residual I²	P-value
**Total**		12	239	-0.13 [-0.24, -0.03]			
**Age**	≤ 24 years	6	118	-0.10 [-0.29, 0.10]	-0.07 [-0.36, 0.22]	40%	0.59
	> 24 years	6	121	-0.17 [-0.39, 0.04]			
**Comparator**	Glucose (1)	7	175	-0.09 [-0.18, 0.00]	1 vs 2: 0.32 [0.06, 0.57]	0%	0.05
	Starch (2)	3	58	-0.41 [-0.65, -0.17]	1 vs 3: -0.04 [-0.42, 0.35]		
	Dextromaltose (3)	2	6	-0.05 [-0.43. 0.32]	2 vs 3: -0.36 [-0.80, 0.08]		
**Dose**	≤ 36 g/day	5	86	-0.24 [-0.45, -0.03]	0.16 [-0.10, 0.43]	30%	0.20
	> 36 g/day	7	153	-0.08 [-0.23, 0.08]			
**Fructose form**	Liquid	10	189	-0.09 [-0.18, 0.00]	-0.32 [-0.57, -0.07]	0%	0.02
	Mixed	2	50	-0.41 [-0.64, -0.18]			
**Design**	Parallel	5	142	-0.20 [-0.40, -0.01]	0.14 [-0.13, 0.40]	34%	0.28
	Crossover	7	97	-0.07 [-0.25, 0.11]			
**Duration**	≤ 2 weeks	8	124	-0.09 [-0.26, 0.09]	-0.12 [-0.42, 0.17]	40%	0.37
	> 2 weeks	4	115	-0.21 [-0.45, 0.02]			
**Energy Balance**	Positive	4	83	-0.07 [-0.30, 0.17]	1 vs 2: 0.10 [-0.26, 0.45]	42%	0.72
	Negative	6	56	-0.16 [-0.43, 0.10]	1 vs 3: 0.13 [-0.26, 0.53]		
	Neutral	2	100	-0.20 [-0.52, 0.12]	2 vs 3: 0.04 [-0.38, 0.45]		

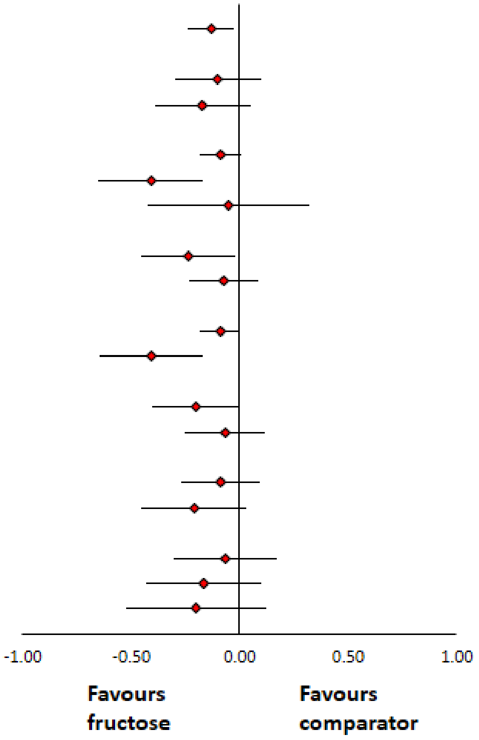

Figure S11. Forest plot of subgroup analyses investigating the effect of small doses of fructose on fasting glucose. Subgroups include age, comparator, dose, fructose form, design, duration and energy balance. Data are represented as MD with 95% CIs. Differences between subgroups were tested using meta-regression and the significance level was reported as a p-value, where $p<0.05$ is considered significant. The residual I^2 value indicates heterogeneity unexplained by the subgroup.

Subgroup	Level	Trials	N	Mean difference (95% CI) in fasting glucose (mmol/L) Within subgroups	Between subgroups	Residual I^2	P-value
**Total**		12	239	-0.13 [-0.24, -0.03]			
**Random Sequence Generation**	Unclear ROB (1)	11	175	-0.14 [-0.29, 0.02]	0.04 [-0.41, 0.49]	41%	0.86
	Low ROB (2)	1	64	-0.10 [-0.52, 0.32]			
	High ROB (3)	0	-	-			
**Allocation Concealment**	Unclear ROB (1)	9	169	-0.15 [-0.33, 0.03]	1 vs 2: 0.05 [-0.44, 0.54]	46%	0.84
	Low ROB (2)	1	64	-0.10 [-0.56, 0.36]	1 vs 3: -0.08 [-0.77, 0.60]		
	High ROB (3)	2	6	-0.02 [-0.53, 0.49]	2 vs 3: -0.13 [-0.67, 0.41]		
**Blinding of Participants and Personnel**	Unclear ROB (1)	5	72	-0.26 [-0.51, -0.02]	0.17 [-0.11, 0.46]	10%	0.07
	Low ROB (2)	7	167	-0.09 [-0.23, 0.05]			
	High ROB (3)	0	-	-			
**Blinding of Outcome Assessment**	Unclear ROB (1)	8	118	-0.20 [-0.39, -0.01]	0.11 [-0.15, 0.38]	28%	0.20
	Low ROB (2)	4	121	-0.09 [-0.27, 0.09]			
	High ROB (3)	0	-	-			
**Incomplete Outcome Data**	Unclear ROB (1)	1	29	-0.11 [-0.47, 0.25]	-0.03 [-0.42, 0.37]	36%	0.83
	Low ROB (2)	11	210	-0.14 [-0.30, 0.03]			
	High ROB (3)	0	-	-			
**Selective Outcome Reporting**	Unclear ROB (1)	0	-	-	-	-	-
	Low ROB (2)	12	239	-0.13 [-0.24, -0.03]			
	High ROB (3)	0	-	-			

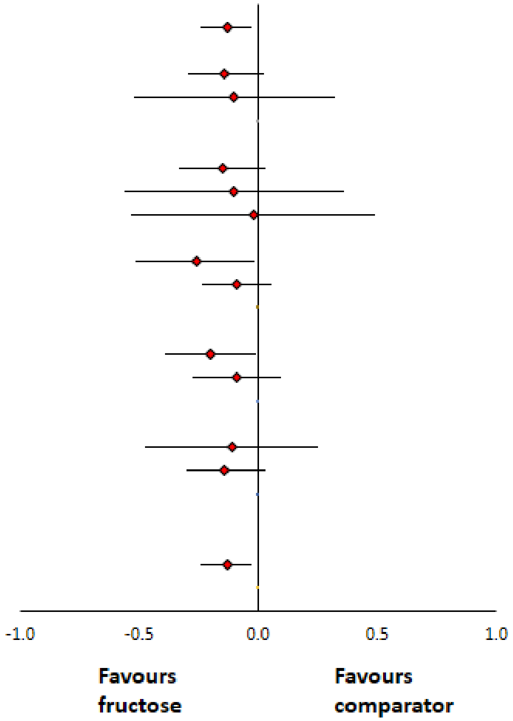

Figure S12. Forest plot of subgroup analyses investigating the effect of small doses of fructose on fasting glucose. Subgroups include random sequence generation, allocation concealment, blinding of participants and personnel, blinding of outcome assessment, incomplete outcome data, and selecting outcome reporting. Data are represented as MD with 95% CIs. Differences between subgroups were tested using meta-regression and the significance level was reported as a p-value, where $p<0.05$ is considered significant. The residual I^2 value indicates heterogeneity unexplained by the subgroup.

Subgroup	Level	Trials	N	Mean difference (95% CI) in fasting insulin (pmol/L): Within subgroups	Mean difference (95% CI) in fasting insulin (pmol/L): Between subgroups	Residual I^2	P-value
**Total**		10	196	2.72 [-4.19, 9.62]			
**Age**	≤ 24 years	6	118	1.87 [-7.58, 11.32]	5.35 [-18.50, 29.20]	5%	0.62
	> 24 years	4	78	7.22 [-14.68, 29.12]			
**Comparator**	Glucose (1)	6	146	5.02 [-5.62, 15.66]	1 vs 2: 3.01 [-14.72, 20.74]	0%	0.30
	Starch (2)	2	44	2.01 [-12.17, 16.19]	1 vs 3: 29.70 [-12.06, 71.46]		
	Dextromaltose (3)	2	6	-24.68 [-65.07, 15.70]	2 vs 3: 26.69 [-16.11, 69.49]		
**Dose**	≤ 36 g/day	4	72	4.54 [-6.52, 15.59]	-4.66 [-22.32, 13.00]	5%	0.56
	> 36 g/day	6	124	-0.13 [-13.90, 13.65]			
**Fructose form**	Liquid	9	160	2.92 [-7.95, 13.80]	-0.84 [-21.05, 19.37]	9%	0.93
	Mixed	1	36	2.08 [-14.95, 19.11]			
**Design**	Parallel	4	128	6.90 [-2.69, 16.50]	-14.76 [-32.78, 3.26]	0%	0.10
	Crossover	6	68	-7.86 [-23.11, 7.40]			
**Duration**	≤ 2 weeks	8	124	0.62 [-7.96, 9.19]	20.28 [-6.37, 46.93]	0%	0.12
	> 2 weeks	2	72	20.90 [-4.33, 46.13]			
**Energy Balance**	Positive	3	54	-4.96 [-22.52, 12.61]	1 vs 2: -7.87 [-30.85, 15.11]	2%	0.48
	Negative	5	42	2.91 [-11.90, 17.73]	1 vs 3: -11.81 [-33.74, 10.13]		
	Neutral	2	100	6.85 [-6.28, 19.98]	2 vs 3: -3.94 [-23.73, 15.86]		

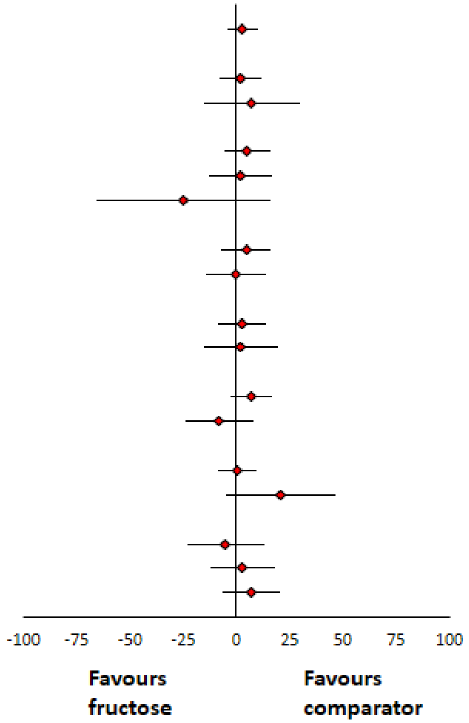

Figure S13. Forest plot of subgroup analyses investigating the effect of small doses of fructose on fasting insulin. Subgroups include age, comparator, dose, fructose form, design, duration and energy balance. Data are represented as MD with 95% CIs. Differences between subgroups were tested using meta-regression and the significance level was reported as a p-value, where $p<0.05$ is considered significant. The residual I^2 value indicates heterogeneity unexplained by the subgroup.

Subgroup	Level	Trials	N	Mean difference (95% CI) in fasting insulin (pmol/L) Within subgroups	Between subgroups	Residual I^2	P-value
**Total**		10	196	2.72 [-4.19, 9.62]			
**Random Sequence Generation**	Unclear ROB (1)	9	132	0.60 [-7.95, 9.15]	21.70 [-5.66, 49.06]	0%	0.11
	Low ROB (2)	1	64	22.30 [-3.69, 48.29]			
	High ROB (3)	0	-	-			
**Allocation Concealment**	Unclear ROB (1)	1	64	1.85 [-7.13, 10.83]	1 vs 2: 20.45 [-7.67, 48.57]	0%	0.13
	Low ROB (2)	7	126	22.30 [-4.35, 48.95]	1 vs 3: 46.98 [-1.40, 95.37]		
	High ROB (3)	2	6	-24.68 [-65.07, 15.70]	2 vs 3: 26.53 [-14.83, 67.90]		
**Blinding of Participants and Personnel**	Unclear ROB (1)	4	58	0.87 [-14.00, 15.75]	2.86 [-14.75, 19.15]	8%	0.73
	Low ROB (2)	6	138	3.73 [-7.52, 14.99]			
	High ROB (3)	0	-	-			
**Blinding of Outcome Assessment**	Unclear ROB (1)	6	104	-2.45 [-12.74, 7.85]	13.68 [-3.08, 30.43]	0%	0.10
	Low ROB (2)	4	92	11.23 [-1.99, 24.45]			
	High ROB (3)	0	-	-			
**Incomplete Outcome Data**	Unclear ROB (1)	0	-	-	-	-	-
	Low ROB (2)	10	196	2.72 [-4.19, 9.62]			
	High ROB (3)	0	-	-			
**Selective Outcome Reporting**	Unclear ROB (1)	0	-	-	-	-	-
	Low ROB (2)	10	196	2.72 [-4.19, 9.62]			
	High ROB (3)	0	-	-			

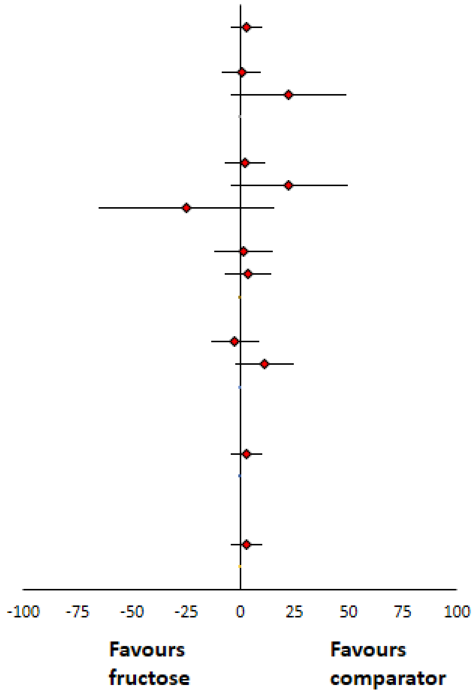

Figure S14. Forest plot of subgroup analyses investigating the effect of small doses of fructose on fasting insulin. Subgroups include age, comparator, dose, fructose form, design, duration and energy balance. Data are represented as MD with 95% CIs. Differences between subgroups were tested using meta-regression and the significance level was reported as a p-value, where p<0.05 is considered significant. The residual I^2 value indicates heterogeneity unexplained by the subgroup.

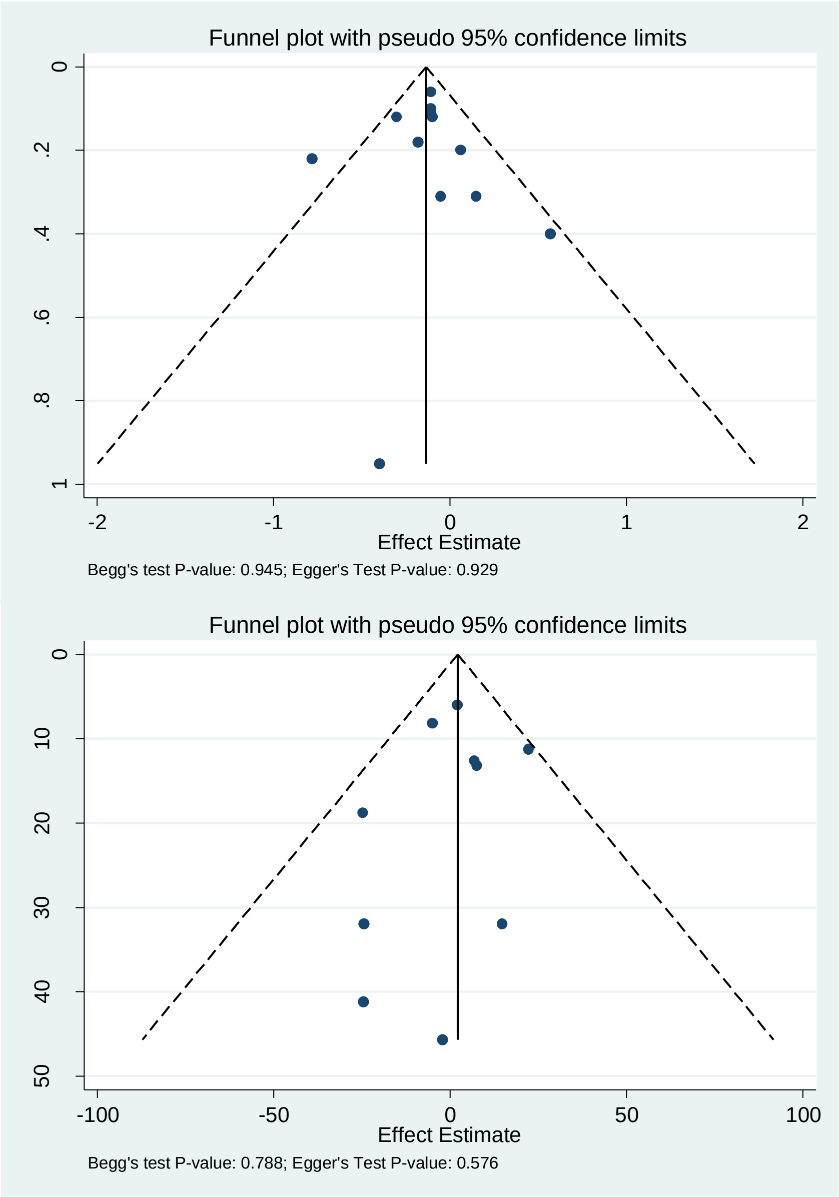

Figure S15. Publication bias funnel plots for the effect of small doses ($\leq$50g/d) of fructose on fasting glucose (top) and fasting insulin (bottom). The solid line represents the pooled effect estimate expressed as the mean difference (MD). The dashed line represents pseudo-95% confidence intervals and the circles represent effect estimates for each included study. P-values were derived from quantitative assessment of publication bias by Egger's and Begg's test set at a significance level of $p<0.05$
